# Supplementary material for: Nursing Students’ Experiences in School-Based Mental Health Promotion: A Qualitative Study in Chile
Source: Nurs Rep. 2025 Nov 29;15(12):427. doi: 10.3390/nursrep15120427 (PMC12736248; doi:10.3390/nursrep15120427)
Supplement: Supplementary file 1 [file nursrep-15-00427-s001.zip › nursrep-3946113-supplementary.pdf]

COREQ Checklist – Page Mapping

Tong A, Sainsbury P, Craig J. Consolidated criteria for reporting qualitative research (COREQ): a 32-item checklist for interviews and focus groups. Int J Qual Health Care. 2007;19(6):349–357.

Domain 1: Research team and reflexivity

Item	Question	Reported on Page No.
1	Which author/s conducted the interview or focus group?	p. 10
2	What were the researcher's credentials?	p. 4
3	Occupation at the time of the study	p. 1 (title page)
4	Was the researcher male or female?	Not reported
5	What experience or training did the researcher have?	p. 4
6	Was a relationship established prior to study commencement?	p. 4
7	What did participants know about the researcher?	p. 4
8	What characteristics were reported about the interviewer/facilitator?	p. 4

Domain 2: Study design

Item	Question	Reported on Page No.
9	Methodological orientation underpinning the study	p. 4
10	How were participants selected?	p. 4
11	How were participants approached?	p. 4-5
12	How many participants were in the study?	p.4
13	How many refused/dropped out?	p. 4
14	Where was the data collected?	p. 4-5
15	Was anyone else present besides participants and researchers?	Not reported
16	What are the important sample characteristics?	p. 4
17	Were interview guides/questions provided? Pilot tested?	p. 4 – no pilot reported
18	Were repeat interviews carried out?	p. 4-5 (no repeat interviews required)
19	Did the research use audio or visual recording?	p. 4-5
20	Were field notes made during/after the interview?	p. 4

21	What was the duration of interviews/focus group?	p. 4
22	Was data saturation discussed?	p. 4
23	Were transcripts returned to participants for comment/correction?	p. 5

Domain 3: Analysis and findings

Item	Question	Reported on Page No.
24	How many data coders coded the data?	p. 5
25	Did authors provide a description of the coding tree?	p. 5-6
26	Were themes derived from the data?	p. 5
27	Was software used to manage the data?	p. 5
28	Did participants provide feedback on the findings?	p. 5 (transcripts returned and validated)
29	Were participant quotations presented to illustrate the themes?	p. 6-7
30	Was there consistency between the data presented and the findings?	p. 7-8
31	Were major themes clearly presented?	p. 7-8
32	Is there a description of diverse cases or minor themes?	p. 7-8
